# Computer Vision-Assisted Data Analysis for Correlative Electron Microscopy and Secondary Ion Mass Spectrometry Imaging

**DOI:** 10.1021/acs.analchem.5c04489

**Published:** 2025-10-12

**Authors:** André du Toit, Alicia A. Lork, Carl Ernst, Nhu T. N. Phan

**Affiliations:** † Department of Chemistry & Molecular Biology, 3570 University of Gothenburg, Medicinaregatan 7B, 413 90 Göteborg, Sweden; ‡ Montreal Neurological Institute, 5620 McGill University, H3A 2B4 Montreal, Canada

## Abstract

Correlative imaging is a powerful analytical approach in bioimaging, as it offers complementary information on the samples measured by different modalities. Particularly, correlative transmission electron microscopy (EM) and nanoscale secondary ion mass spectrometry (NanoSIMS) imaging enable high-resolution morphological and chemical analysis at the subcellular level. However, manual segmentation and correlation of regions of interest (ROIs) in large EM and NanoSIMS data sets are time-consuming, prone to user bias, and limited in throughput. To address this, we developed a computer vision-assisted image analysis pipeline for automatic classification and segmentation of subcellular organelles in EM images, enabling rapid and reproducible correlation with NanoSIMS ion data. Using human neuronal progenitor cells (hNPCs) and differentiated postmitotic neurons, we trained a YOLOv8 deep learning model to recognize six major organelle types. The pipeline included EM image preprocessing, segmentation via YOLOv8, morphological filtering, and image registration with NanoSIMS ion maps. Performance evaluation demonstrated a robust model accuracy. We applied the pipeline to measure ^15^N-leucine abundance to study protein turnover in single organelles across different cell states. Results showed distinct turnover dynamics among organelles, with slower turnover observed in differentiated neurons compared to hNPCs. The automated pipeline significantly reduced the analysis time (from hours to minutes) while maintaining consistency with manual segmentation. Our approach demonstrates how computer vision can streamline correlative imaging workflows, improve data quality, and enable deeper insights into subcellular processes such as protein turnover, making it especially valuable for SIMS users and broader bioimaging applications.

## Graphical abstract

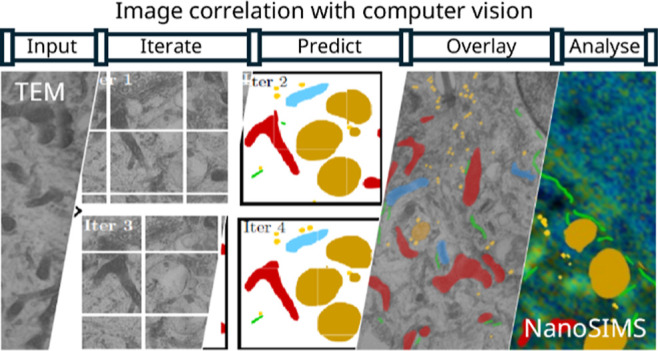

## Introduction

Over the past decade, computer vision has become increasingly prevalent in research and across a wide range of industries. It involves the use of computers to extract meaningful information from image data, emulating the capabilities of human vision. Computer vision principles rely on extracting relevant features and patterns from images or videos and using them to infer meaning and make decisions through hierarchical architectures.^1^ This includes a wide range of tasks, particularly image classification, object detection, segmentation, tracking, and pattern recognition. These tasks can be applied in numerous applications and fields, such as autonomous vehicles,^2^ robotics,^3^ medical imaging,^4^ electron microscopy,^5^ as well as mass spectrometry (MS) data.^6,7^

A wide variety of bioimaging techniques generate data that, when correlated with other techniques, provide greater context and information about regions of interest (ROIs), revealing relationships that cannot be observed with a single modality. Data correlation, however, can be extremely time-consuming and burdensome for the users, and it can introduce inherent bias in demarcating ROIs by the users that affects the reproducibility. Moreover, the development of new advanced instruments capable of resolving ultrastructures in biological research has led to the generation of mega-images that can be difficult to work with and interact with. It is therefore necessary to be able to handle and process large data sets using highly efficient image analysis approaches that are capable of automation and standardization.

For image analysis in electron microscopy (EM), convolutional neural networks among other approaches, are used for recognition and classification of nanostructures, for example, nanoparticles in material sciences^5,8^ or cellular organelles in biology.^9,10^ The task of classification involves assigning classes, such as types of organelles in the case of life sciences, to detected objects based on their features or attributes. It can be used in image processing to distinguish regions of interest by assigning a label to each pixel in a process known as segmentation. In life sciences, computer vision has been applied for the classification and segmentation of organelles, such as mitochondria^10^, as well as whole-cell organelle segmentation in volumetric electron microscopy data.^11^ An advantage of whole-cell organelle segmentation is that it helps identify multiple types of ROIs within a single image. ROI selection based on organelle labeling is significantly limited to one or two types of organelles in some advanced imaging techniques, especially fluorescence microscopy. On the other hand, EM, with an ability to visualize morphological features of subcellular compartments at a nanometer spatial resolution, makes it possible to perform whole-cell organelle segmentation in one image.

EM can be combined with other modalities to obtain complementary information on the studied samples. Correlation between EM and nanoscale secondary ion mass spectrometry (NanoSIMS) imaging helps characterize the subcellular morphological structures and chemical organization of tissues and cells.^12^ This is very useful for understanding the structural and functional relation that underlies biological processes, especially for NanoSIMS and other SIMS that cannot identify cellular and subcellular structure alone. In brief, NanoSIMS employs a high-energy primary ion beam (Cs^+^ or O^–^) to sputter the sample surface, generating secondary ions (mostly monatomic or diatomic ions) from the samples. These secondary ions are then extracted into the mass spectrometer for separation by a magnetic sector mass analyzer and detection based on their specific mass per charge (*m*/*z*). Up to seven detectors are available, providing up to seven ion images of the sample surface obtained in parallel. NanoSIMS has been used to visualize and relatively quantify the distribution of elements and isotopes in biological samples at a subcellular spatial resolution, which is compatible with EM imaging.^13^ Thicker sample sections required for NanoSIMS decrease electron permissibility, resulting in low signal-to-noise ratio images, making it challenging to identify specific structures, or ROIs, in EM images in a consistent manner. This is particularly problematic when a large number of ROIs or multiple types of ROIs need to be identified in large image data sets. In addition, manual ROI selection can be a laborious and time-consuming process that, when processing a large image data set in combination with inherent variation in ROI selection, is a poor cost-effective approach.

Here, computer vision is an ideal solution owing to its efficient classification and segmentation of ROIs for correlative EM/SIMS images in an objective, nonbiased, and high-throughput manner. The process is potentially automated, which significantly improves the quality of the results and the efficiency of the workload. There are different models of computer vision that can be utilized depending on the complexity, size, and nature of the given data and task; for example, one has been used successfully for particle selection in CryoEM.^14^ However, considering the unique challenges of EM data due to the sample preparation required for NanoSIMS that was addressed above, a suitable model would need to be trained with the original EM data obtained using the same sample preparation and imaging conditions as for the analysis data to ensure consistency and accuracy in the ROI selection at a nanoscale level.

In this project, we develop a computer vision-assisted pipeline for automatic, robust classification and segmentation of ROIs on EM images for correlation with NanoSIMS images using YOLO (you only look once),^15^ a popular computer vision model that can be used for object detection, image classification, and instance segmentation tasks. We demonstrate the utility of the computer vision-assisted image analysis in the identification of multiple types of organelles in single neuronal cells imaged by correlative EM and NanoSIMS. This will serve as a pragmatic tool for users in the SIMS community who are not familiar with the computational field to obtain an accurate, objective, and high-throughput data analysis process for image correlation with EM. Application of the analysis pipeline to protein turnover analysis revealed distinct turnover dynamics across organelles at different neuronal stages, highlighting its significant potential for SIMS users and broader utility in advanced bioimaging studies.

## Methods

### Cell Culture and Sample Preparation

Human neuronal progenitor (hNPC) cells were obtained from the Carl Ernst lab, McGill University, Montreal, Canada. The use of these human cells was approved by the Research Ethics Board of the McGill University Health Center with ethics approval code 23-09-075 and the date of approval of December 9, 2024. hNPCs were maintained in cell culture dishes (MatTek, #P35G-1.5-14-C) coated with poly-l-lysine (Sigma-Aldrich, #A-004) and laminin (Thermo Fisher Scientific, #23017015) with STEMdiff NPC medium (StemCell Technologies, #05833) supplemented with 200 ng/mL Sonic Hedgehog at 37 °C in a humidified atmosphere supplemented with 5% CO_2_. To differentiate hNPCs into postmitotic neuronal cells, hNPCs were maintained for 1 weeks in BrainPhys Neuronal Culture Medium (STEMCELL technologies, #05790) supplemented with 2% B27 (Thermo Fisher Scientific, #17504044), 1% N_2_ (Thermo Fisher Scientific, #A1370701), 20 ng/mL BDNF (Genescript, #Z03208), 20 ng/mL GDNF (Genescript, #Z03387), 200 nM ascorbic acid (STEMCELL technologies, #72132), 1 mM dibutyryl cAMP (STEMCELL technologies, #100-0244), 1 μg/mL laminin. For the experiments of protein turnover in hNPCs and postmitotic neuronal cells, cell culture medium was incubated with media supplemented with 2 mM ^15^N-leucine for 48 h (pulse period). Thereafter, cells were rinsed and maintained in fresh culture medium without ^15^N-leucine and maintained for 0, 12, 24, 48, and 96 h (chase period). A control group was also included that was pulsed for 2 days with the culture medium without ^15^N-leucine.

After the chase period, cells were washed with prewarmed dPBS (Thermo Fisher Scientific, #14190144) and fixed with prewarmed Karnovsky fixative (2.5% glutaraldehyde (Sigma-Aldrich, #G6257), 2% paraformaldehyde (Sigma-Aldrich, #P6148), and 0.02% sodium azide (Sigma-Aldrich, #S2002) at pH 7.4.) in 0.1 M sodium cacodylate buffer for 20 min at room temperature (RT). This was followed by washing six times with PIPES solution for 5 min each at RT. The fourth washing step included 50 mM glycine (Sigma-Aldrich, #G7126) to block unreacted aldehydes. Thereafter, cells were fixed with 1% osmium tetroxide (Sigma-Aldrich, #201030) in PIPES solution for 30 min on ice in the dark.

Samples were washed six times with MiliQ water for 3 min, followed by fixation with 1% uranyl acetate (Electron Microscopy Sciences, #541-09-3) in water for 30 min on ice. Samples were then washed three times with water and subsequently dehydrated with 30, 50, 70, 85, 95, and 100% ethanol (5 min each, three times 100% ethanol) on ice. Afterward, samples were infiltrated with Agar100 resin (Agar Scientific, #AGR1031) in a 1:2 ratio in 100% ethanol for 15 min at RT, followed by 2:1 resin to 100% ethanol for another 15 min. Eventually, cells were infiltrated three times with 100% Agar100 (5, 5, and 10 min at RT). Finally, cells were incubated with Agar100 containing accelerator BDMA for 10 min and mounted onto a filled gelatin capsule (Agar Scientific, #AGG29209), followed by polymerization at 60 °C for 16 h. Resin-embedded samples were cut into sections of 150 nm thickness using a Leica UC7 ultramicrotome. The sections were then placed on TEM finder grids (Electron Microscopy Sciences, #FCF200F1-Cu).

### TEM Imaging

EM images of the cell sections were acquired on a Talos L120C G2 transmission electron microscope (Thermo Fisher Scientific) equipped with a LaB6-source, which was operated at 120 kV, and a Ceta CMOS camera, using MAPS software (Thermo Fisher Scientific, MAPS2) for automated image acquisition at X11 000 magnification.

### NanoSIMS Imaging

NanoSIMS measurements were performed on the cell areas which had been imaged with TEM using a 16 keV Cs^+^ primary ion source. A primary ion fluence of 3 × 10^16^ Cs^+^ cm^–2^ was implanted on the sample surface prior to each measurement. The images were acquired with a primary ion current of 0.9–1 pA, a diaphragm D1–4 (150 μm width), and a dwell time of 5 ms. The entrance slit was selected at 20 μm width, the aperture slit was at 200 μm width, and the energy slit was fully open, providing the mass resolving power up to 7000 for ^12^C^14^N^–^ ion at *m*/*z* 26.003. The pixel size was kept at around 78 nm per pixel. Each image was obtained with 4–5 image planes reaching a fluence of at least 4 × 10^15^. Detectors were set to detect the secondary ions: ^12^C_2_^–^, ^12^C^14^N^–^, and ^12^C^15^N^–^, which will be simplified as ^12^C_2_, ^12^C^14^N, and ^12^C^15^N in the following sections.

### Image Correlation and Data Analysis

EM images were stitched using MAPS software (Thermo Fisher Scientific) and processed using a Python-scripted pipeline consisting of several Python packages (Table 1).

**Table 1:** Python Packages Included in the Python-Scripted Pipeline

package (version)	purpose	ref	web site
Python 3.9.17	computing platform	^16^	www.python.org
Numpy 1.26.0	numerical computing	^17^	www.numpy.org
OpenCV 4.8.0	image processing	^18^	www.opencv.org
Skimage 0.21.0	image processing	^19^	www.scikit-image.org
Ultralytics (YOLOv8)	deep learning and computer vision	^20^	www.ultralytics.com

### Training Deep Learning YOLO Model

A training data set was built from EM images in which the ROIs for each type of organelle are demarcated based on their morphological features, representing nucleolus, mitochondria, endoplasmic reticulum (ER), Golgi complex, vacuoles, and vesicles (Figure 1) to create ROI masks that correlate with EM images. EM image contrast was adjusted by matching the histogram to a reference image (selected based on the best contrast), padded, and cut into a matrix grid of smaller images with a frame dimension of 640 × 640 pixels (the largest input frame size of the YOLO model). Images and the corresponding ROI masks were ordered according to the Ultralytics hierarchical path structure and were used to train a YOLOv8 extra-large segmentation model (YOLOv8x-seg.pt) for 250 epochs, where one epoch is one complete pass of the training data set through the algorithm, accelerated with a GPU (GeForce RTX 2070).

**Figure 1:**
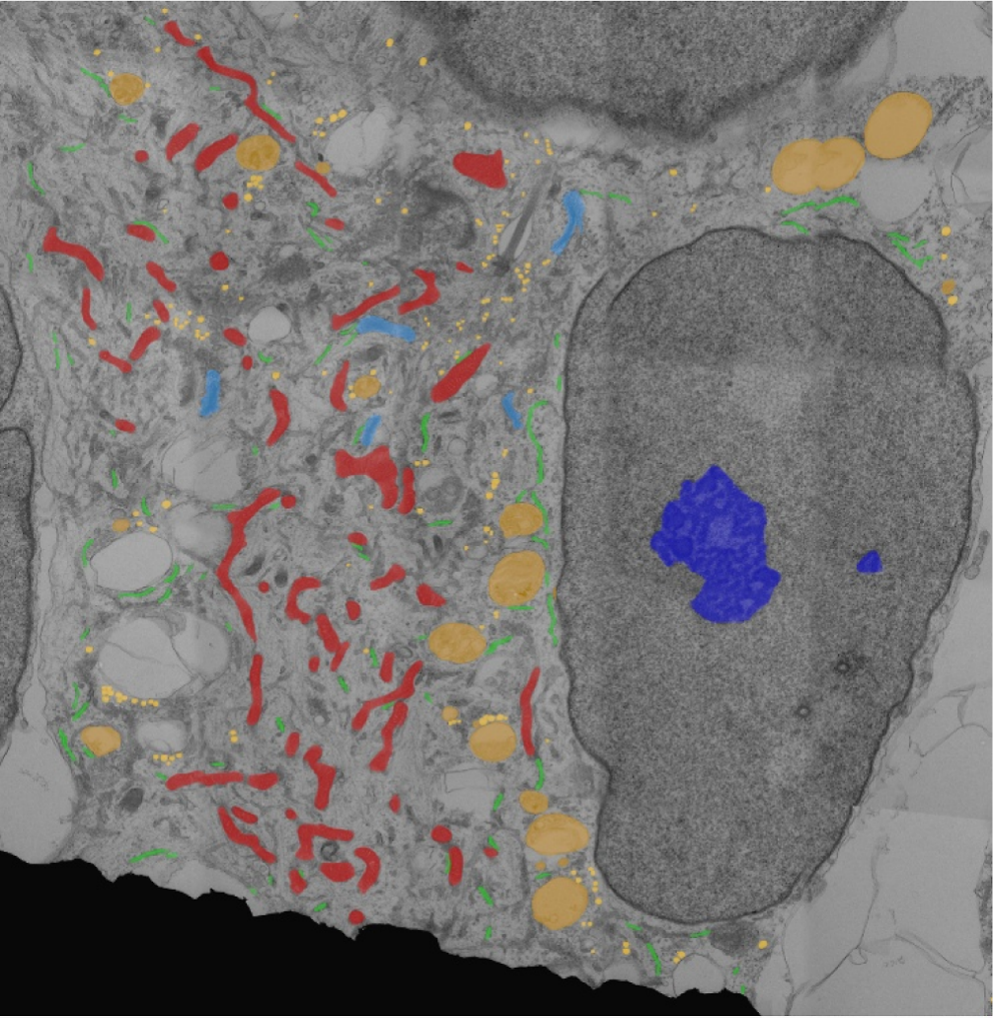
Example image of an overlay obtained by manually ROI segmentations on a TEM image of an hNPCs cell (gray scale image) used for training the YOLO model. ROIs: nucleolus (■-dark blue), mitochondria (■-red), endoplasmic reticulum (■-green), Golgi complex (■-light blue), vacuoles (■-orange), and vesicles (■-yellow).

### Automated Image Segmentation

Histograms of the input EM images were matched to those of the reference EM images used in training, then padded, and cut into a matrix grid consisting of smaller sections of the input image, each section with a dimension of 640 × 640 pixels. To allow multiple iterations of the input image to ensure sufficient coverage of ROIs for automated segmentation, a series of matrices were generated, each matrix offset from the previous matrix (an example in Figure 2). These smaller sections of the input image were parsed through YOLO predict to segment ROIs in the image frames. Segmented ROIs were then compiled into a single mask for the input image frame to correlate with the NanoSIMS image. This was followed by morphological analysis and filtering to remove partially identified ROIs, for example, to remove partially detected vesicles based on their circular shape.

**Figure 2:**
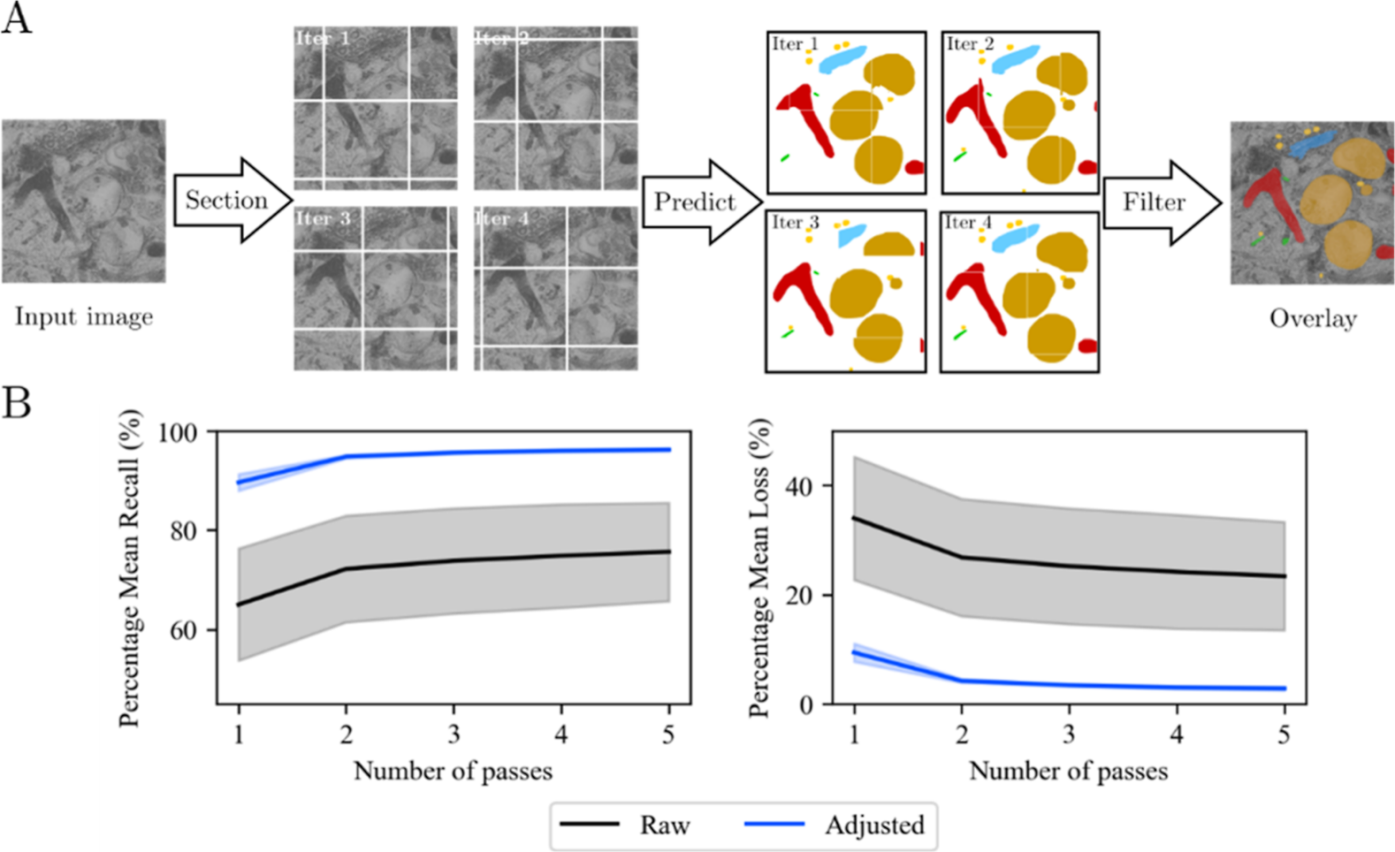
ROI segmentation pipeline scheme using computer vision. (A) Input TEM image of hNPCs is segmented into multiple matrices, each overlapping the previous, allowing multiple passes over ROIs. By parsing segmented images through the YOLO predictor, a single reconstructed image is compiled, followed by morphological filtering. ROIs; mitochondria (■-red), endoplasmic reticulum (■-green), Golgi complex (■-light blue), vacuoles (■-orange), and vesicles (■-yellow). (B) Impact of multiple passes on ROI segmentation accuracy. Raw refers to the original TEM image input, while adjusted refers to preprocessed TEM images (via histogram matching). The solid lines show the mean recall (left), i.e., the proportion of true positive values correctly predicted, and the mean loss (right), i.e., incorrectly predicted cases. Gray and blue shading areas indicate the standard deviation.

### Manual ROI Segmentation and Multipoint ROI Selection

ROIs were manually segmented using Affinity Photo (www.affinity.serif.com). ROIs were also selected using the ‘multi-point’ function in FIJI^21^ to generate a list of coordinates of the respective organelles that was used to retrieve isotopic counts for the respective ion species.

### EM and NanoSIMS Image Correlation

A simple graphic user interface was scripted using the Tkinter library^22^ in Python to manually overlay EM images with NanoSIMS images of the same cell areas. This allows recording the translocation, rotation, and scale parameters of the respective images for automated overlay of the segmented EM images and the NanoSIMS ones. The raw SIMS data were imported into a Python program using Sims 2.0.2 (www.pypi.org/project/sims/), a module to read Cameca SIMS data files in Python, then were proceeded with drift and dead-time corrections, followed by a correlation of the automated segmented ROI masks with the NanoSIMS ion images.

## Results and Discussion

### Correlative EM/NanoSIMS Image Data Analysis and Challenges

Correlation of EM and NanoSIMS images requires multiple data analysis platforms, including several steps; the overlaying between EM and NanoSIMS images, then an ROI selection/segmentation on the EM images, followed by matching the ROI position onto the NanoSIMS images to extract the chemical information on individual ROIs from the NanoSIMS data. The conventional approach is to perform manually the ROI segmentation and correlation with NanoSIMS images. This is a very tedious and time-consuming task, especially when many different ultrastructures of the samples are desired. From our previous experience, this ROI segmentation and correlation could take around half a day for a single EM image to detect six different types of ROIs (this also depends on the number of ROIs desired in the image). Moreover, the inherent variability in ROI segmentation, resulting from bias in analysis, data analysts’ experience, and variation between samples, also compromises the reproducibility of the data analysis. Given the simplicity, versatility, and scalability within a single platform, computer vision allows low-biased and automated selection of ROIs once being integrated into image analysis, facilitating correlation with NanoSIMS data quickly and accurately.

### Neural network–driven Detection

You Only Look Once (YOLO) is a computer vision algorithm for real-time object detection. It directly predicts bounding boxes/segmentation mask and class probabilities from an image in a single pass through a neural network (Figure S1A), unlike older methods that perform object detection as a two-step process, including a generation of regional proposals (regions that the YOLO model proposes to contain the objects) and region classification. YOLO partitions the input image into multiple grids (80 × 80, 40 × 40, and 20 × 20), where each grid is tasked with detecting objects whose center lies within its boundaries. Each grid predicts a fixed number of segmentations (e.g., 3–5 per grid cell depending on the YOLO version) and a set of class probabilities (e.g., nucleolus, mitochondria, endoplasmic reticulum). For the final prediction, the confidence score is multiplied by class probabilities, giving a per-class confidence for each bounding segment. In overlapping predictions, YOLO applies non-maximum suppression (NMS) to remove duplicate segments. This keeps the segment with the highest confidence and suppresses others with high overlap (IoU threshold). YOLO uses a convolutional neural network (CNN) as a feature extractor (EfficientNetBackbone model in YOLOv8). The CNN produces a feature map that is parsed to the detection heads (a part of the YOLO framework that takes the feature maps to generate the final predictions) that predicts segments and class assignments at multiple scales, allowing for the detection small and large objects.

### Creating Training Data

To train a YOLO segmentation model, the user must first create a data set that accurately represents the desired ROIs. This involves preparing the corresponding input images and label files, structured according to the YOLO-recognized data set format, followed by the creation of a data set configuration file and the subsequent execution of the training process (Figure S1B–E). YOLO does not accept mask images directly; instead, it requires a specific segmentation label format, in which the boundary coordinates of each ROI are stored in a text file with the corresponding class number. The simplest approach is to generate binary mask images of the ROIs and then convert these masks into label files in the required format. For this conversion, we employed the cv.findContours function in a Python script. The data set is organized into three folders: train, validation (val), and test, which are used for model training, hyperparameter tuning, and performance evaluation, respectively. Note that the test folder is optional and only required if an independent evaluation data set is available; otherwise, the performance can be assessed using the validation set alone. A data set configuration file (.yaml) specifies data set-specific parameters, including paths to the train/val/test data sets, class names, and the number of classes. Model training is initiated from the command line using the YOLO command, where the user defines the training task (e.g., segmentation), the pretrained model to be used (here, yolov8x-seg.pt), the location of the configuration file, the number of epochs, and the image size. Numerous additional parameters are also available for fine-tuning.

The amount of required training data is dependent on several factors, including sample diversity, ROI type, and morphological distinctiveness. As a starting point, approximately 20% of the total EM images were manually annotated and used for training, which was sufficient for most ROIs, particularly those with distinct morphologies, such as mitochondria. For more challenging structures, such as the Golgi apparatus, a larger proportion of the data set (30%) was included to ensure accurate detection. Here, roughly 40 cells were used for training (the total number of cells imaged was approximately 180). The overarching goal is to generate a robust model capable of generalizing across diverse cell morphologies. Once established, such a model can be applied in future studies without the need for retraining, provided that the morphological features remain consistent.

Training images were preprocessed prior to model development, which included adjusting contrast. Contrast often varies largely among EM images, especially when images are acquired in an automated manner using the same acquisition parameters. Besides, variation in sample thickness and cell staining also influences the contrast level. Therefore, preprocessing can play a key role in standardizing image histograms to enable a more reliable automated analysis. Newer EM imaging systems may incorporate advanced acquisition protocols that reduce contrast variability, potentially minimizing the need for preprocessing.

### Adapting to Large Images

TEM image dimensions are several orders of magnitude larger than the input dimensions of the YOLO model. Parsing a large image would, by default, bin (rescale) the image to match the model dimension, resulting in a potential loss of image information. This would make it difficult to recognize subcellular morphological features, such as mitochondria, in a binned mitochondrial image with a few tens of pixels compared to a highly resolved image with several hundreds of pixels due to the loss of topological information from binning. The solution to this problem involves segmenting a large TEM image into a matrix and parsing every frame individually to preserve the high resolution and information on the image. It is, however, possible that ROIs may be truncated by this method, which impacts both the training of the model and the detection of ROIs. Therefore, it is recommended to perform multiple passes on a large EM image by sectioning it into several matrices, each offset from the previous, so that a combined ROI mask sufficiently reflects the ROIs (Figure 2). Here, a pass refers to a single iteration, where the model processes one matrix of the image, and multiple passes ensure that overlapping regions are analyzed more than once. In this case, each matrix was offset by 128 pixels to obtain five iterable matrices (obtained by dividing the number of desired iterations by the model frame’s input dimensions, 640 pixels), allowing multiple overlapping passes over ROIs to improve recall accuracy (Figure 2B).

### ROIs Segmentation and Validation

The trained YOLO model was readily used to identify specific morphological features that correspond to various types of cellular organelles. With the model, a single EM image was processed within several minutes, significantly accelerating the analysis speed. To validate the segmentation results, several performance metrics, particularly mean average precision (mAP), intersection over union (IoU), recall, precision, and F1 score, can be employed to examine the efficiency and accuracy of the object detection/segmentation results. The IoU measures the overlap between a predicted segmented mask and the ground truth, where the ground truth is the manually segmented ROIs used in setting up the training data. The data set is divided into training, validation, and testing subsets. The validation data set is used to tune and validate the model during training, while the testing data set is reserved for final evaluation. An IoU of 1 indicates a perfect overlap, and 0 indicates no overlap. The IoU is most often used as a threshold to decide if a predicted segmented mask is considered to be true positive (e.g., IoU = 0.5). Precision refers to how many predicted positives are actually correct, and it is expressed between 1 and 0, where 1 indicates being correct at 100%. It gives the users the information on the precision degree of the model. Recall measures how many actual positives were correctly predicted. F1 Score is the harmonic mean of precision and recall that integrates precision and recall in a single score. The mAP is the mean of area under the precision–recall curve across all classes and is the most common metric in object detection tasks. The greater the mAP value, the better the model performs at both finding objects (recall) and making correct predictions (precision). They provide a valuable insight into the YOLO model’s performance and how to improve it. Further information on these metrics and their implications can be found in relevant documents (YOLO, https://docs.ultralytics.com/). Figure 3A demonstrates the YOLO model’s performance for each epoch iteration for EM segmentation (an epoch is a complete pass of the training data set through the algorithm), improving after each iteration; and an increase in the accuracy of the segmentation is indicated by the mAP trend toward 1.0. This indicates that the predictions almost perfectly match the ground truth across all evaluated classes. It is noted that overfitting can lead to inaccurate predictions due to the model’s inability to generalize. To avoid overfitting or underfitting, it is important to find the right balance between model complexity and the amount and diversity of training data. YOLO provides the best-fitting weights as output, making it easier for scientists with little machine learning experience to apply the most suitable parameters to their data. Another useful tool to assess the model’s performance is the confusion matrix, which compares the predicted classes against the ground truth within a matrix (Figure 3B). In the confusion matrix, the *x*-axis represents the correct organelles as manually identified, and the *y*-axis represents the predicted organelle output by the YOLO model. Each number in the matrix indicates the relative frequency of predictions for the corresponding correct-predicted pair of organelles. The values in the right column denote the fraction of background regions that were misclassified as a given organelle, while the values in the bottom row denote the fraction of organelles that were misclassified as background. Thus, the most common error by the model is organelles being classified as background rather than another organelle type. This is likely due to low contrast boundaries between organelles and the surrounding cytoplasmic content in EM images. A classwise breakdown of the performance metrics shows that mitochondria and nucleoli were detected with the highest accuracy due to their distinct and easily recognizable morphological features compared to other organelles.

**Figure 3:**
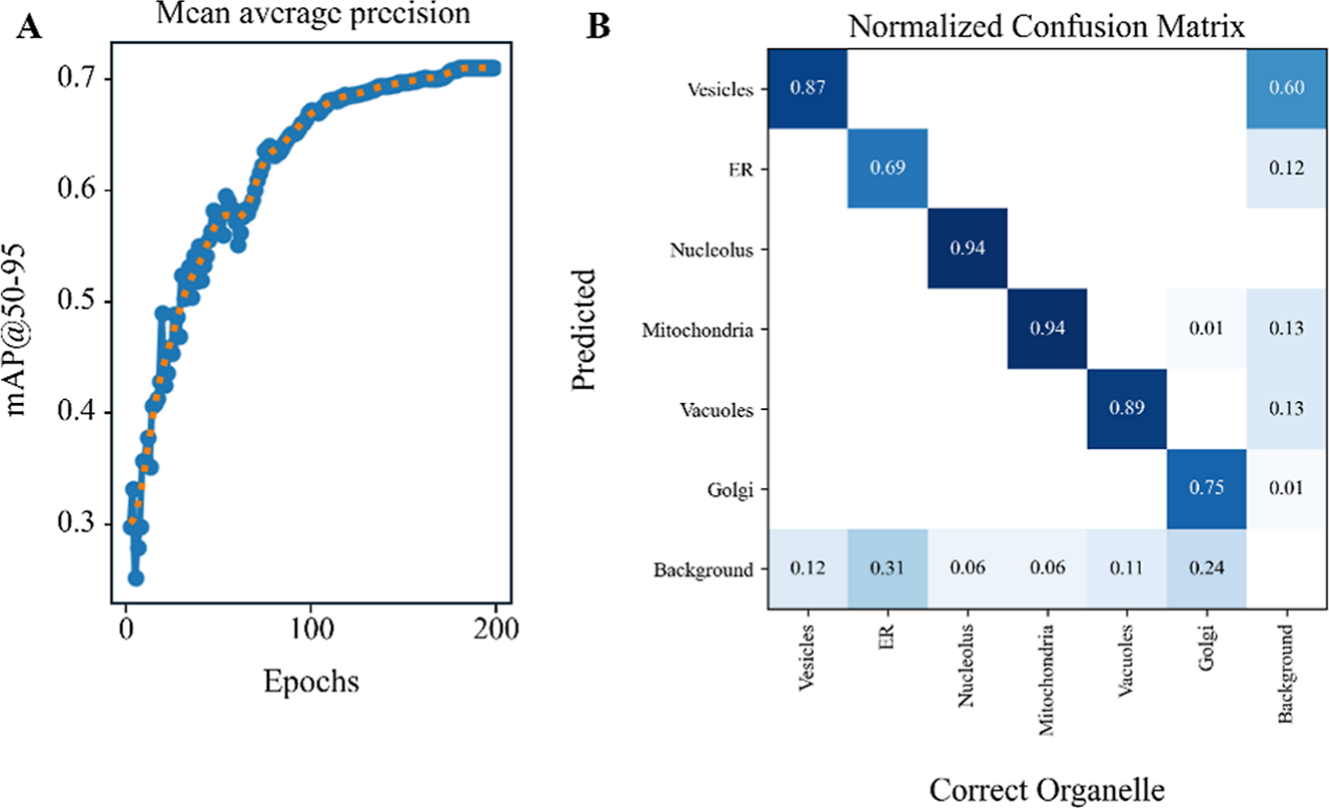
Performance metrics used for evaluating the YOLO machine learning model. (A) Mean average precision, an accuracy metric for segmentation performance for each iteration that the training data set has completely passed through the algorithm (epoch) during training. The mean average precision (mAP) is the mean of the area under the precision–recall curves across all classes (e.g., nucleus, mitochondria, etc.), with the intersection over union (IoU) threshold set between 0.5 and 0.95 (m@AP0.5–0.95). (B) Visualization and quantification of the proportion of correct and incorrect predictions (background) for the respective cellular organelles. The *x*-axis represents the correct organelles as manually identified, while the *y*-axis represents the predicted organelles by the model. Each value within the matrix corresponds to the relative frequency of predictions for the given correct–predicted pair of organelles.

### Image Analysis Pipeline

With Python, image processing algorithms, machine learning algorithms, and numerical/statistical analysis can be combined into a single image segmentation and correlation pipeline, including image preprocessing, automated segmentation, image correlation, and data extraction. The first step in the image analysis involves image pretreatment, which improves performance and quality significantly. In this step, adjusting the image contrast results in an improvement of approximately 20% in the accuracy of ROI segmentation (Figure 2B). There are useful algorithms, including histogram matching, equalization, and contrast stretching to automatically normalize image brightness and contrast. Here, histogram matching (from scikit) was applied to transform the input image so that its histogram matched that of a reference image exhibiting optimal contrast. Following image pretreatment, ROIs can be efficiently segmented based on normalized image data using the automated segmentation and evaluation method described above. The next step is to overlay EM and NanoSIMS images, which can be done manually by identifying cell structures or semiautomatically using anchor points in both EM and NanoSIMS images. Here, a simple graphic user interface was utilized to manually overlay EM images with NanoSIMS images to obtain translocation, rotation, and scale parameters, which are used to correlate ROI mask and isotopic NanoSIMS data. Images were warped so that the EM image of a cell was geometrically stretched and aligned to match the same cell as imaged by NanoSIMS. Image registration can be used to spatially transform EM images and, consequently, ROI masks to align with NanoSIMS images using a set of common anchor points defined manually. Alternatively, image registration can be done by using the anchor points that are generated automatically by specific feature extraction, albeit with some limitations. For instance, cellular nuclei that are automatically segmented in both EM and NanoSIMS images can be used as the anchor points to register the rotation, scaling, and translocation parameters and for image correlation. To correlate NanoSIMS image data with ROI masks, the raw SIMS data are imported into Python and then drift and dead-time corrected, followed by correlation between the ROI mask and the individual NanoSIMS ion images. With the aim to determine the protein turnover and protein lifetime within the cellular organelles of interest as an example of application in our study, we incorporated an exponential decay model fitting into the analysis pipeline and used it in statistical analysis. The image analysis pipeline developed here represents a highly reproducible, efficient, and nonbiased approach to dissecting complex image data, enabling the SIMS users to obtain accurate correlative chemical and morphological information at a sub-cellular resolution. This is particularly very useful for SIMS applications in biology and life science, where large data sets are often handled. The pipeline can be scripted for automation to facilitate image analysis within the SIMS community.

### Utilizing Computer Vision-Assisted Data Analysis to Investigate Subcellular Protein Turnover in hNPCs and Postmitotic Neuronal Cells

Protein turnover is a critical cellular process representing the replacement of old proteins with newly synthesized ones. This process is highly regulated to maintain an intact cellular proteome, ensuring proper cellular functions. Protein turnover has been shown to be closely related to cellular functions at the subcellular level. Particularly, protein turnover was found to correlate with synaptic activity at single synapses in rat-cultured hippocampal neurons.^23^ In addition, our previous study reported that the protein turnover is highly heterogeneous at a single organelle level in human neural progenitor cells.^12^ This emphasizes the need to study the subcellular spatial organization of protein turnover to further understand the functional regulation of protein turnover in cellular systems at the organelle level.

Protein turnover has been investigated by different analytical techniques, such as fluorescence microscopy and mass spectrometry.^24–26^ NanoSIMS has been used to study the protein turnover of organelles such as in synapses,^23^ lysosomes,^27^ and stress granules^24^ due to its capability of high spatial resolution imaging (≈50 nm), high mass resolution (*m*/Δ*m* ≈ 10,000), and good sensitivity (ppb–ppm detection limit).^28^ The protein turnover can be measured using a pulse–chase labeling approach, in which cells are first incubated with isotopically labeled (e.g., ^13^C^–^, ^15^N^–^) amino acids, allowing their incorporation into newly synthesized proteins (pulse phase). The cells are then incubated in a nonisotopic cell medium for a period of time during which the isotopically labeled proteins are gradually declining in abundance due to a replacement by nonisotopic newly synthesized proteins (chase phase). By tracking the enrichment of the isotopes over the chase period, the protein turnover rate and the half-life of the proteins can be determined. High-resolution NanoSIMS imaging allows visualization of the localization and relative quantification of isotopic species at the subcellular resolution, thereby providing both spatial and semiquantitative information on protein turnover at single organelles.

Correlative EM and NanoSIMS imaging allows the identification of many different organelles and their protein turnover information, enabling a comparison of molecular organization and activity across different types of organelles within single cells. In this section, we performed the imaging of subcellular protein turnover in hNPCs using EM and NanoSIMS correlation and employed the developed analysis pipeline in image analysis. Cell culture, sample preparation, and correlative TEM and NanoSIMS imaging were carried out similarly to the workflow described in the previous study.^12^ The obtained data were processed by the manual analysis and computer-assisted analysis pipeline; the turnover results were compared to confirm the reliability of the computer vision-assisted data analysis.

Using the image analysis pipeline above, organelles from a TEM image were automatically identified and segmented and then correlated with NanoSIMS ion images of the same cell areas within a few minutes (Table 2). Figure 4 shows representative images of correlative NanoSIMS and TEM images of hNPCs for identifying the nucleolus, mitochondria, and Golgi apparatus. On the other hand, manual hand-drawn ROI segmentation took approximately 5 h for each TEM/NanoSIMS image based on our own personal experience using images of 50 × 50 μm field of view with a pixel dimension of ≈2 nm (256 × 256 pixels). Multipoint ROI selection using ‘click tools’ from FIJI was also used for comparison, where ROIs are selected by clicking on specific points in the TEM images for respective organelles. In the NanoSIMS images, the subcellular protein turnover of the cells was indicated via the distribution of the ^15^N enrichment (^12^C^15^N/^12^C^14^N) across the cells. To observe the turnover rate of subcellular regions, we examined the ^15^N enrichment of each region at five time points during the chase period between 0 and 96 h. Figure S2 shows the protein turnover rates of six different cellular organelles, including ER, nucleolus, Golgi complex, mitochondria, vacuoles, and vesicles, over a chase period of 96 h. The turnover rates were shown to follow a first exponential decay trend, which is consistent with previous studies.^12,26^ From the exponential decay equation of the turnover rate, a half-life value, *t*_1/2_, can be calculated to obtain insight into the lifetime of total proteins associated with specific organelles. We have performed a comparison of the performance between the automated ROI segmentation, manual hand-drawing segmentation, and multipoint ROI selection using “click tools”. It was shown that the automated ROI segmentation performance is comparable to the manual hand-drawn segmentation in determining the half-lives of organelles (difference within 1.2–6.2%). On the other hand, the accuracy of multipoint ROI selection using “click tools” ranged between 1.2% and 24.0%, depending on the type of organelles (Table 2) and the number of organelles selected (Figure S4). The large variation in accuracy is possibly due to the type of organelles and the specific location of the click tool in the organelle, as signal heterogeneity exists across the ROI. Both automated and multipoint ROI selection methods required minimal time for ROI segmentation/identification; however, the automated approach required no input from the user beyond the machine learning stage, which makes it the best method for high-throughput data analysis.

**Table 2:** Comparison of Analysis Performance in the Protein Half-Lives (*t*_1/2_) and Analysis Time for Three Selected Types of Organelles Using Manual Hand-Drawing ROI Segmentation, Automatic Computer Vision Assisted ROI Segmentation and Multi-Point ROI Selection

	manual hand-drawn ROI segmentation	automated ROI segmentation	multipoint ROI selection
*t*_1/2_ nucleolus	16.4 ± 0.5 h	16.7 ± 0.7 h	16.5 ± 1.0 h
*t*_1/2_ mitochondria	21.5 ± 1.0 h	20.2 ± 1.1 h	15.8 ± 1.2 h
*t*_1/2_ Golgi	16.4 ± 0.7 h	15.9 ± 0.9 h	16.2 ± 0.5 h
analysis time	≈0.5–5 h^a^	≈2 min	≈5 min

a Depends on the number of ROIs and image size.

**Figure 4:**
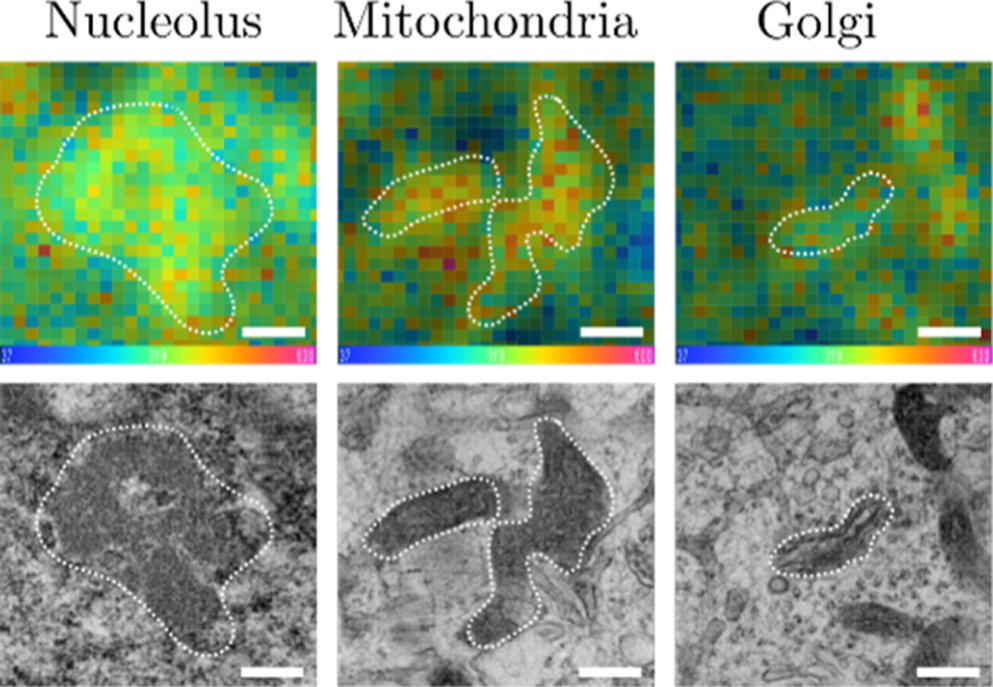
Correlation of representative NanoSIMS ratio images of ^12^C^1^ ^5^N/^12^C^14^N (top row) and TEM (bottom row) images showing nucleolus, mitochondria, and Golgi apparatus in single hNPCs. ROI segmentation was obtained by the automatic analysis pipeline. Scale bars: 250 nm.

### Subcellular Protein Turnover and Protein Lifetime of hNPCs and Postmitotic Neuronal Cells

We measured the subcellular protein turnover in hNPCs and postmitotic neuronal cells using our developed analysis pipeline, examining the six different cellular organelles (Figures 5 and S3). There are significant differences in the protein turnover compared with that between organelles. In hNPCs, mitochondria exhibited the slowest turnover rate and thus the longest protein lifetime (*t*_1/2_ = 21.5 h), followed by vesicles (*t*_1/2_ = 20.2 h) and the ER (*t*_1/2_ = 19.2 h). In contrast, the nucleolus and Golgi apparatus showed the highest turnover rates with the shortest protein lifetimes (*t*_1/2_ = 16.4 h). In postmitotic cells, vesicles exhibited the lowest initial ^15^N enrichment and the longest protein lifetime (*t*_1/2_ = 76.2 h), followed by mitochondria (*t*_1/2_ = 31.6 h), while the ER maintained a relatively short lifetime (*t*_1/2_ = 19.8 h). Comparing across the two cell states, hNPCs displayed a more uniform turnover rate with the half-lives between 16.4 and 21.5 h, whereas postmitotic cells showed a broader distribution of turnover rates, with *t*_1/2_ values ranging from 19.8 to 76.2 h. This narrow range in hNPCs may reflect their similar proliferative activity and need for a dynamic proteome remodeling across the examined organelles to maintain developmental plasticity. In contrast, the greater variation in protein turnover among postmitotic cells, despite comparable sample sizes, could be attributed to their higher degree of cellular differentiation.

**Figure 5:**
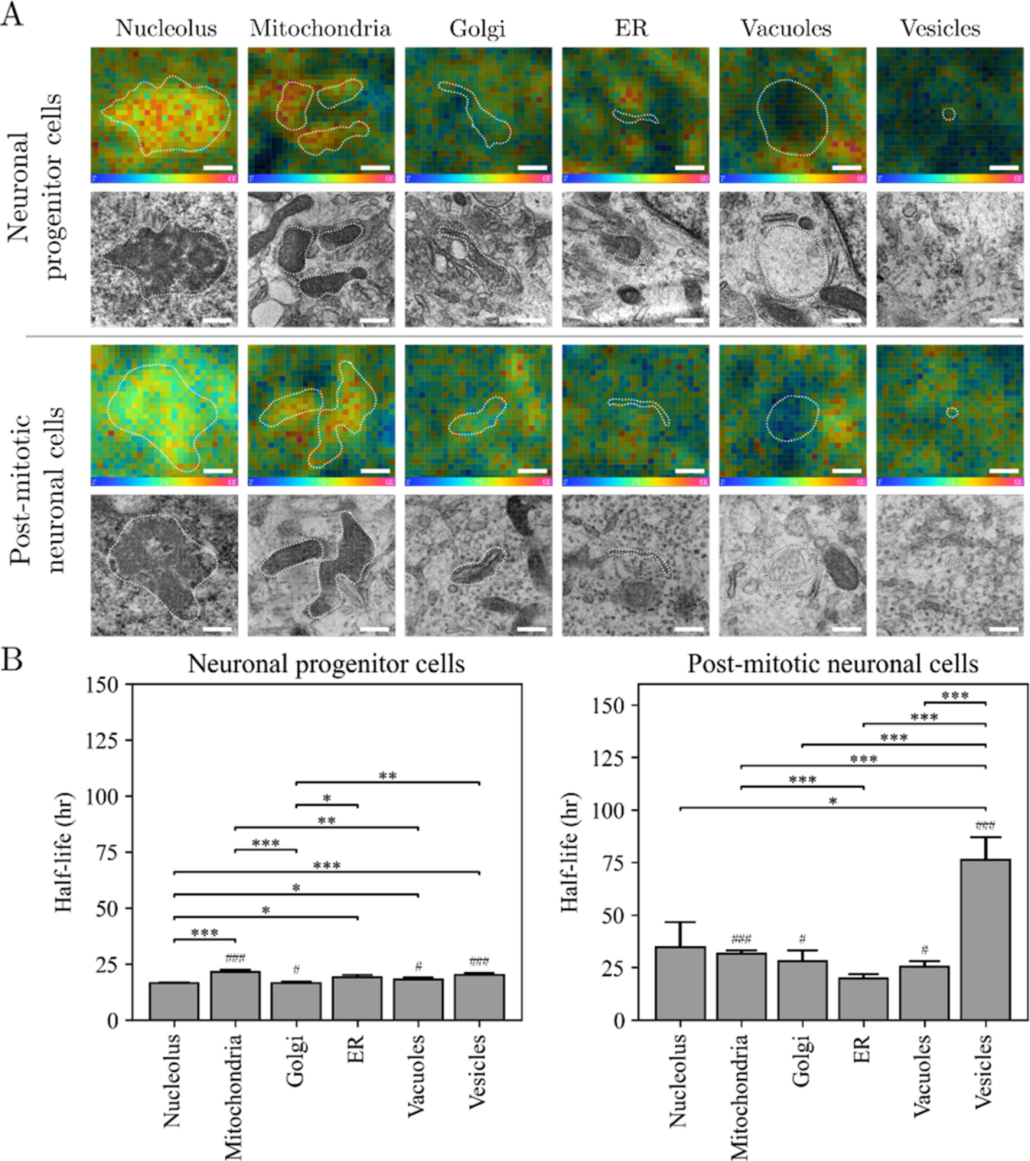
Protein lifetime across cellular organelles in hNPCs and postmitotic neuronal cells imaged by NanoSIMS/TEM correlation. (A) Representative NanoSIMS ratio images of 12C15*N*/12C14N (top row) and TEM (bottom row) images of the organelles in the cells incubated with ^15^N-leucine for 48 h and chased for 24 h. ROI segmentation was obtained by the automatic analysis pipeline. Scale bars: 250 nm. (B) Protein half-lives, *t*_1/2_, within the respective organelles. Error bars are SEM. Significance comparison was performed using an independent *t*-test implemented in SciPy, and significance levels are indicated above (*p < 0.05, **p < 0.01, ***p < 0.001). # indicates significance levels between hNPC and postmitotic neuronal cells regarding a respective organelle (#p < 0.05, ##*p* < 0.01, ###p < 0.001).

Most organelles exhibited a general reduction in protein turnover, meaning a higher protein lifetime in the postmitotic cells. Mitochondria, which play central roles in energy production and the regulation of neurogenesis,^29^ showed increased protein lifetimes in postmitotic neuronal cells. This is consistent with a previous study using ^13^C-lysine labeling in mouse brains, where mitochondrial proteins exhibited extended lifespans exceeding 10 days.^12,25^ The increased mitochondrial lifetime in neurons may reflect lower metabolic demands or altered energy requirements. Vesicles exhibited the greatest increase in protein half-lives in postmitotic neuronal cells, which could be related to a low activity of neuronal secretion, meaning a low need of protein turnover, in this cell state as they are not fully matured neurons. Interestingly, the ER turnover remained relatively stable across both cell stages. Given the ER’s critical functions in protein and lipid biosynthesis, calcium storage, and intracellular signaling, its consistent turnover rate supports the maintenance of essential neuronal processes.^30^ Moreover, ER-mediated calcium regulation is vital for synaptic transmission, and disruptions in this system can have severe consequences for neuronal function. The data revealed significant differences in protein turnover rates at subcellular compartments and between cell stages, providing key insights into how the molecular dynamics at the organelle level contribute to overall proteome maintenance and cellular function.

### Concluding Remarks

In this paper, we designed an image analysis pipeline for correlative EM and NanoSIMS imaging, employing computer vision to automate image segmentation and correlation. The performance of the automatic pipeline was compared with the manual analysis method, demonstrating comparable accuracy while considerably reducing processing time and minimizing the need for supervision. This is potentially a powerful tool for large data image analysis, replacing manual and labor-intensive segmentation of ROIs while assuring the quality of the results in a significant time-saving manner. The application of the pipeline to explore the subcellular protein turnover of hNPCs demonstrates the reliability, reproducibility, and efficiency of the method. This will serve as a very useful data analysis tool to explore sample complexity with multiple parallel information. While the current application is focused on NanoSIMS and EM, the general analysis workflow has a high potential for broader use within the mass spectrometry community, especially in life science. In this wider context, correlation with other established structural imaging technologies, such as SEM, TEM, and H&E staining, has become standardized. The analysis workflow with automated ROI selection and efficient handling of large data sets could provide significant value to the community, enhancing analytical throughput and facilitating comprehensive investigation in life science research.^12,13,31–33^

## Supplementary Material



## Notes

Overview of YOLOv8 setup and usage, including YOLOv8 setup and usage, ^15^N enrichment kinetics in organelles using manual, automated, and multipoint ROI methods, comparison of enrichment kinetics in hNPCs and postmitotic neurons, and impact of ROI selection on ^15^N enrichment analysis (PDF)

## Abbreviations

Cs^+^: cesium primary ion
dPBS: Dulbecco’s phosphate-buffered saline
ER: endoplasmic reticulum
GUI: graphical user interface
hNPC(s): human neuronal progenitor cell(s)
IoU: intersection over union
kV: kilovolt
mAP: mean average precision
*m*/*z*: mass-to-charge ratio
NanoSIMS: nanoscale secondary ion mass spectrometry
O^–^: oxygen primary ion (NanoSIMS)
OpenCV: open source computer vision library
pA: Picoampere
ROI(s): region(s) of interest
SciPy: scientific Python library
SEM: scanning electron microscopy
SIMS: secondary ion mass spectrometry
TEM: transmission electron microscopy
ToF-SIMS: time-of-flight secondary ion mass spectrometry
YOLO: you only look once (real-time object detection framework).
