# Supplementary material for: Computer Vision-Assisted Data Analysis for Correlative Electron Microscopy and Secondary Ion Mass Spectrometry Imaging
Source: Anal Chem. 2025 Oct 12;97(41):22807–16. doi: 10.1021/acs.analchem.5c04489 (PMC12547856; doi:10.1021/acs.analchem.5c04489)
Supplement: Supplementary file 1 [file ac5c04489_si_001.pdf]

Computer vision assisted data analysis for correlative electron microscopy and secondary ion mass spectrometry imaging.

André du Toit,† Alicia A. Lork,† Carl Ernst,‡ and Nhu T.N. Phan†

†Department of Chemistry & Molecular Biology, University of Gothenburg, Medicinaregatan 7B, 413 90 Göteborg, Sweden

‡ McGill University, Montreal Neurological Institute, Montreal, H3A 2B4, Canada

ABSTRACT: Correlative imaging is a powerful analytical approach in bioimaging as it offers complementary information of the samples measured by different modalities. Particularly, correlative transmission electron microscopy (EM) and nanoscale secondary ion mass spectrometry (NanoSIMS) imaging enables high-resolution morphological and chemical analysis at the subcellular level. However, manual segmentation and correlation of regions of interest (ROIs) in large EM and NanoSIMS datasets are time-consuming, prone to user bias, and limited in throughput. To address this, we developed a computer vision-assisted image analysis pipeline for automatic classification and segmentation of subcellular organelles in EM images, enabling rapid and reproducible correlation with NanoSIMS ion data. Using human neuronal progenitor cells (hNPCs) and differentiated post-mitotic neurons, we trained a YOLOv8 deep learning model to recognize six major organelle types. The pipeline included EM image pre-processing, segmentation via YOLOv8, morphological filtering, and image registration with NanoSIMS ion maps. Performance evaluation demonstrated robust model accuracy. We applied the pipeline to measure ^{15}N-leucine abundance to study protein turnover in single organelles across different cell states. Results showed distinct turnover dynamics among organelles, with slower turnover observed in differentiated neurons compared to hNPCs. The automated pipeline significantly reduced analysis time (from hours to minutes) while maintaining consistency with manual segmentation. Our approach demonstrates how computer vision can streamline correlative imaging workflows, improve data quality and enabling deeper insights into subcellular processes such as protein turnover, making it especially valuable for SIMS users and broader bioimaging applications.

Table of content:

Figure S1: Overview of YOLOv8 setup and usage. YOLOv8 setup, architecture, dataset format, label structure, command-line usage.

Figure S2: The time-dependent change of the ^{15}N enrichment for different cellular organelles over 96 h of chase time using manual, automated, and multi-point ROI segmentation.

Figure S3: The time-dependent change of the ^{15}N enrichment for different organelles in hNPCs and post-mitotic neuronal cells over 96 h of chase time analyzed by automated pipeline.

Figure S4: Relationship between the number of organelles selected using the multi-point ROI selection tool and the resulting relative ^{15}N enrichment via the $^{12}C^{15}N/^{12}C^{14}N$ ratio to show effects of manual organelle point selection on the relative ^{15}N enrichment.

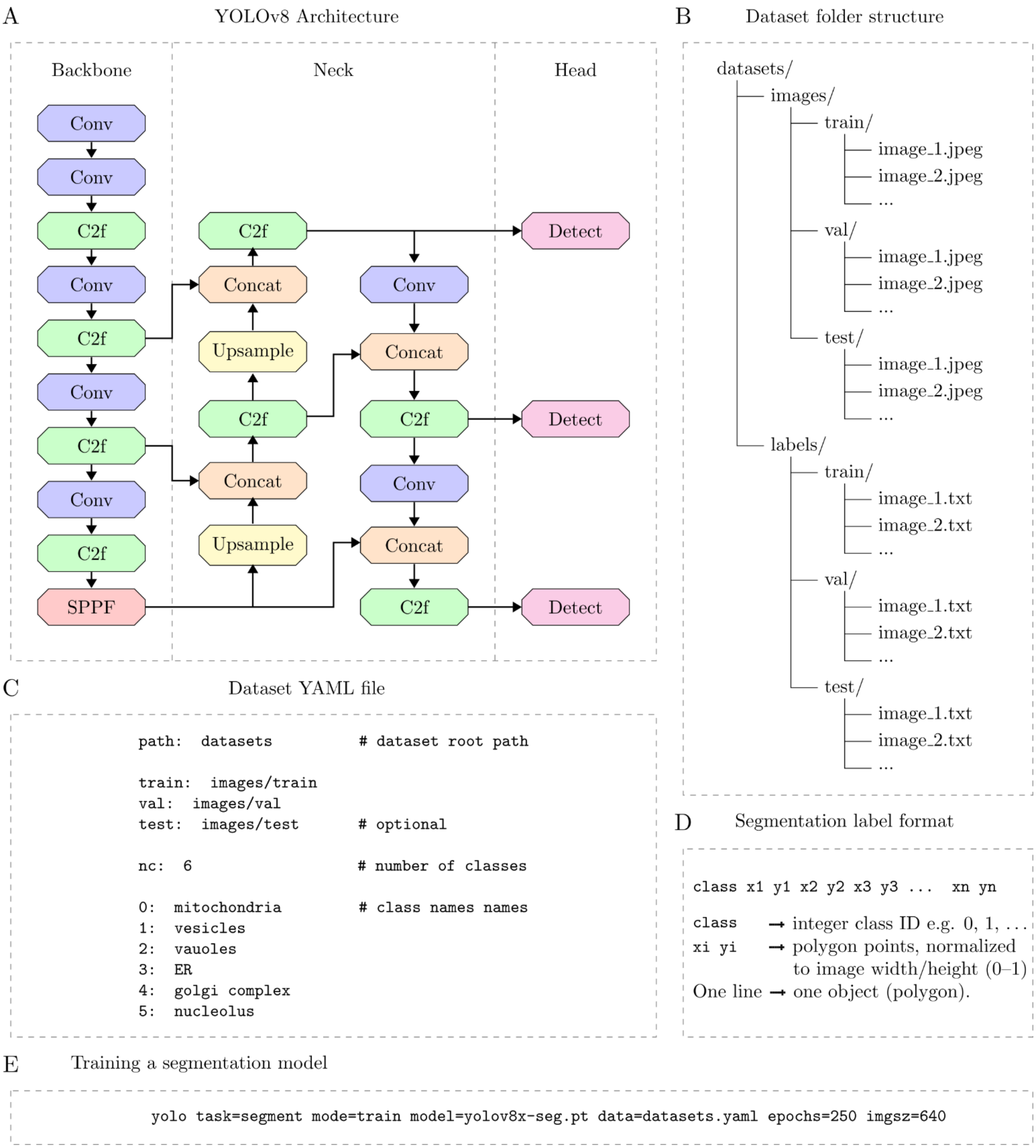

Figure S1: Overview of YOLOv8 setup and usage. A) Simplified YOLOv8 CNN architecture. The backbone is built from Conv layers and C2f modules, which split features into two paths (Conv and neck/detect) and then fuse them with Concat operations to improve gradient flow and efficiency. The Neck fuses multi-scale features via Upsampling and lateral connections, while the Detect head predicts classes, and segmentation masks. B) Required dataset folder structure with images/ and labels/ sub- directories split into train/, val/, and optionally test/. Each image is paired with a corresponding label text file describing the ROI mask. C) Example of a dataset YAML file, which defines the dataset paths, number of classes (nc), and class names. D) Segmentation label test format of ROIs. Each object is represented as class_id x1 y1 x2 y2 ... xn yn, where polygon coordinates are normalized between 0–1 relative to image size. One line corresponds to one object. E) Example usage in command line, showing how to train a segmentation model with a chosen architecture (yolov8x-seg.pt) and a custom dataset YAML file.

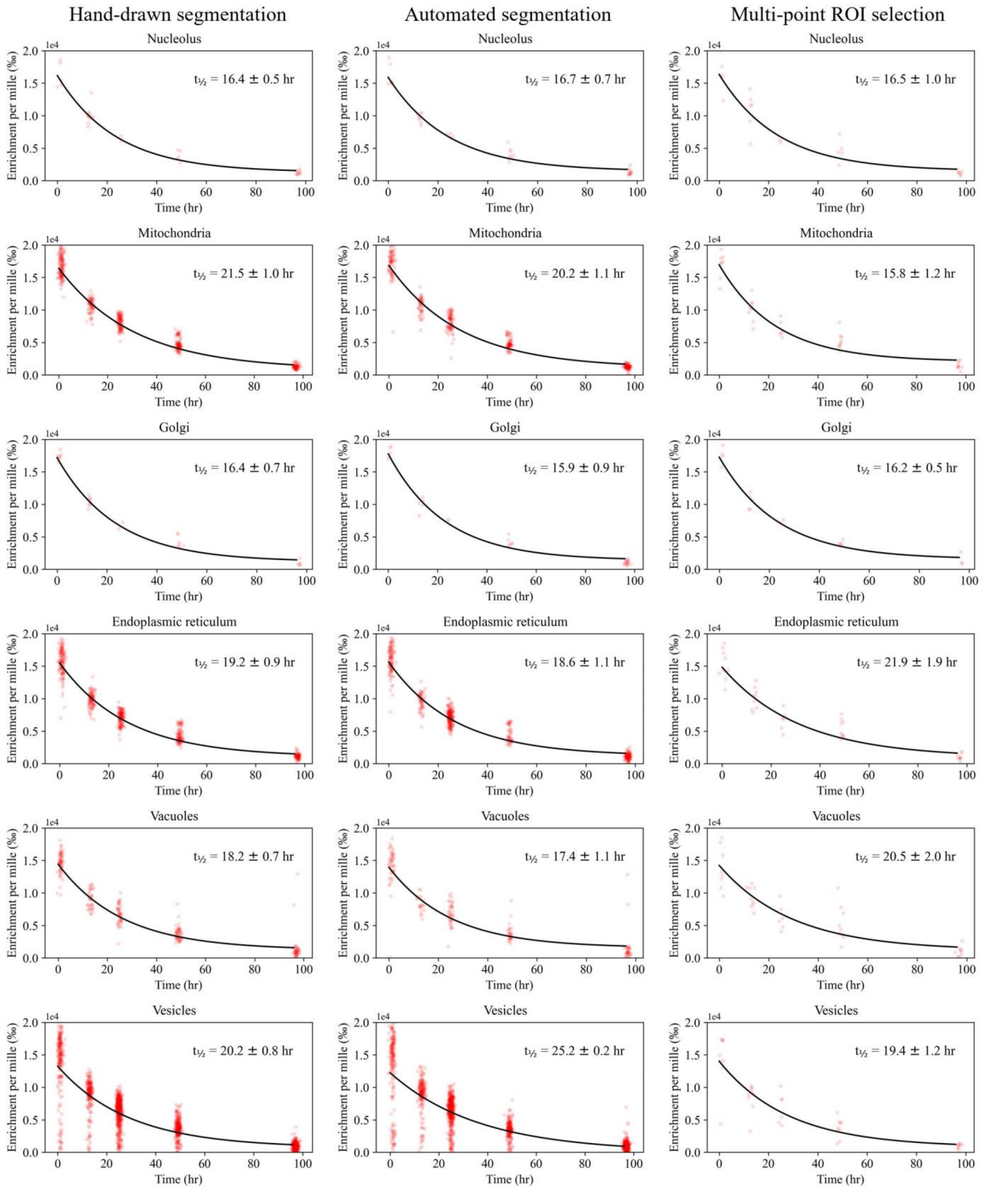

Figure S2: The time-dependent change of the ^{15}N enrichment for different cellular organelles over 96 h of chase time. Organelles were identified using manual hand-drawn segmentation (left column), automated segmentation (center column), and multi-point ROI selection (right column). A black line represents the first exponential decay-fitted model to the data, and the protein half-life is indicated on the graph as $t_{½}$.

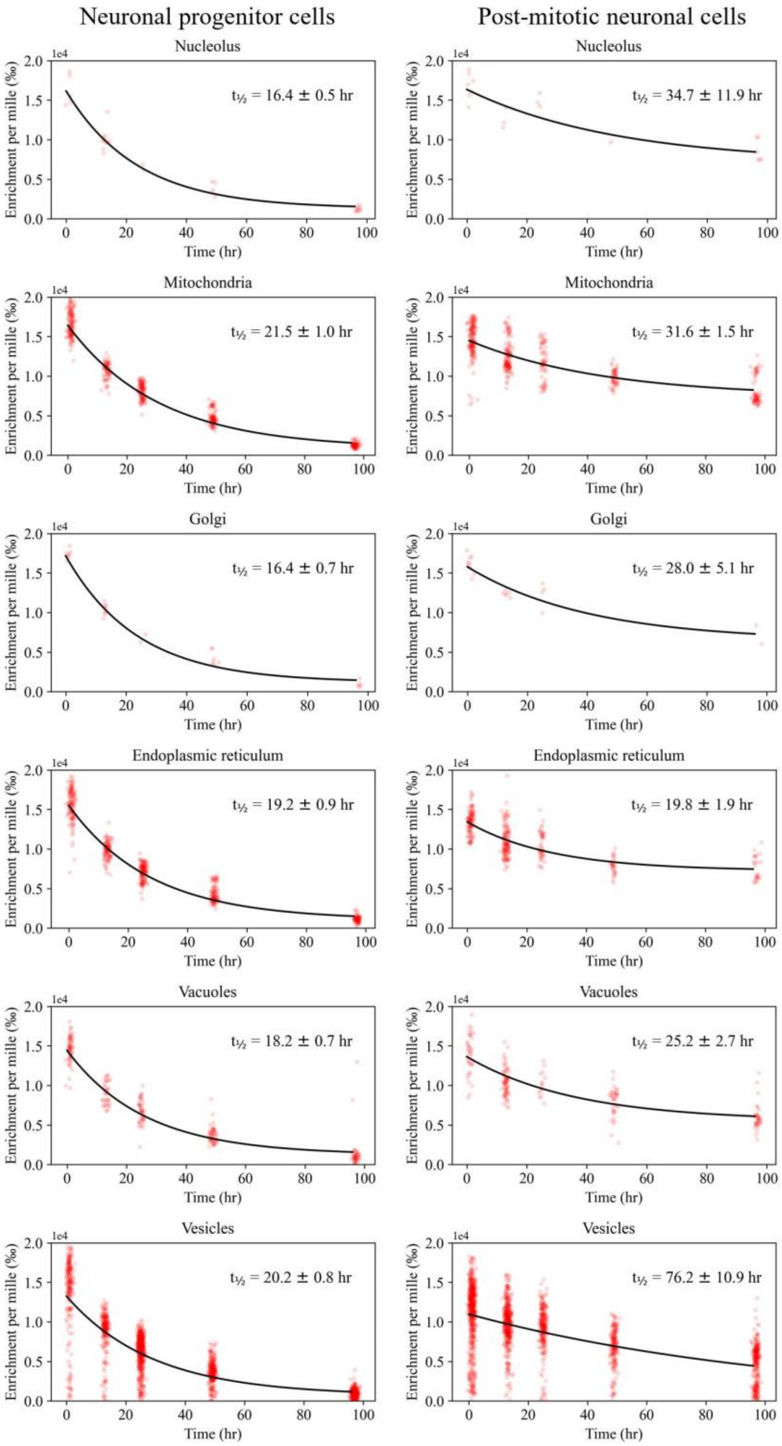

Figure S3: The time-dependent change of the ^{15}N enrichment for different organelles in hNPCs (left) and post-mitotic neuronal cells (right) over 96 h of chase time. Image data were proceeded using the automated analysis pipeline. A black line represents the first exponential decay-fitted model to the data, and the protein half-life is indicated on the graph as $t_{½}$.

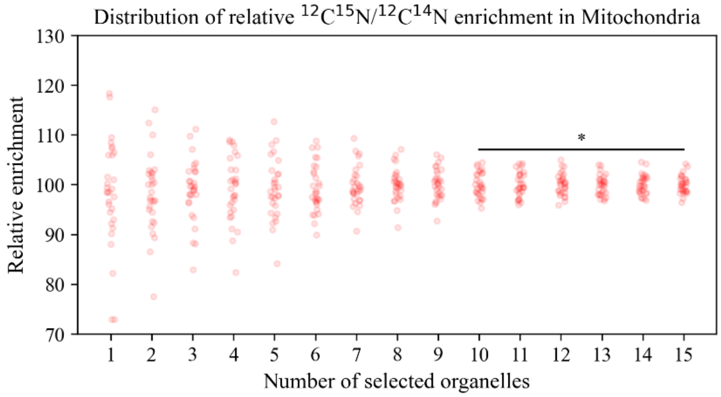

Figure S4: Relationship between the number of organelles selected using the multi-point ROI selection tool and the resulting relative ^{15}N enrichment via the $^{12}C^{15}N/^{12}C^{14}N$ ratio. The aim was to determine the minimum number of ROIs (or organelles) needed to achieve reliable and consistent enrichment measurements. For each selection size, the process was repeated 10 times on 3 separate images (30 measurements per data point) to simulate repeated analysis attempts and to assess variability. An F-test was performed to evaluate differences in variance in relative enrichment across selection sizes. For mitochondria, selecting at least 10 organelles per image resulted in the most consistent and robust enrichment values (* $p < 0.05$).
